# The Week's Work

**Published:** 1909-10-30

**Authors:** 


### The Week's Work.

THE event of the week in hospital circles has been the visit of His Majesty the King to Norwich, where he laid the foundation-stone of the new isolation block of the Norfolk and Norwich Hospital. A report of the proceedings appears elsewhere in this issue, and we refer to it here to draw attention to the interesting and thoughtful reply which His Majesty made to the loyal address presented to him on the occasion of his visit. In that reply the King pointed out the debt which the Norwich Hospital owes to the President of its Board of Management, Lord Leicester, who has taken a warm and singularly active interest in the work of the institution, and has been largely instrumental in evoking public support in aid of the new extensions. Referring to hospital work in general, His Majesty reiterated his feelings of cordial interest in hospital work in this country, of which he is known as one of the staunchest admirers and friends, and especially expressed his approval of the scheme under which the institutional nurses are encouraged to join the Nursing Service of the Territorial Forces. The King's visit and words of appreciation will be worth much to the Norfolk and Norwich Hospital, which is to be congratulated upon starting the practical realisation of its hopes so auspiciously. The new isolation block is the practical commencement of a scheme which was adopted in October 1908 for increasing the efficiency of the hospital. This scheme, which was based on a report of the honorary medical staff, involves an estimated outlay of £50,000, half of the amount for buildings and half for endowments. The only accommodation at present for infectious cases is two rooms on the ground floor of the old wing of the hospital. The new block, which is designed for eight beds, will be built on a site some distance from the main building. With its equipment it will cost £3,059. The septic block will accommodate ten beds for surgical cases of such a nature that they could not be treated in the ordinary surgical wards without a danger of infection to other cases. The cost and equipment of this block will be £2,575.

LAST week the Lord Mayor of Liverpool issued a circular letter suggesting the formation of a Liverpool Central Council of Charity to organise local charitable relief. There are many organisations in Liverpool for the relief of distress, but it appears that there is a good deal of waste and extravagance in dealing with the contributions which reach them. We do not think Liverpool stands solitary in deploring the want of a central advisory and managing council or board to deal with charitable relief; but the Lord Mayor's enthusiasm is highly commendable, and it is to be hoped that his suggestions will receive practical attention. It will be remembered that the recently issued report of the Royal Commission on the Poor Laws advised the formation of local central committees which should act conjointly with the various local organisations in dealing with the distribution of voluntary and official relief. Such central bodies are already at work in several foreign and Continental cities, and we have in mind the charitable central councils which are to be found in Dresden, Berlin, and Breslau as cases in point. The Liverpool scheme, as outlined by the Lord Mayor, provides for the proper representation on the Central Council of Charity for the city of both the Local Government Board and the various religious denominations. It also provides for the establishment of a certain station or clearing house, which can deal with all forms of relief as occasion arises. The scheme is an ambitious one, and there is no reason why it should not be successfully carried out now that the Poor Law Commissioners' Report has drawn public attention to the necessity for alterations in some forms of relief work. It is a suitable time for trying to evoke some practical interest in the matter which the Lord Mayor of Liverpool has so enthusiastically taken up.

THE Asylum Officers' Superannuation Bill, to which we referred last week, reached the Committee stage in the House of Lords on Monday, and gave rise to some interesting discussion. The Earl of Donoughmore moved two amendments to the first clause of the Bill to provide that established officers and servants should be divided into two classes for the purposes of superannuation. He mentioned that the amendments were really submitted in the name of the London County Council, who had ten asylums under its charge. The County Council contemplated the addition of another asylum, and about 20,000 patients were under its care. The result was that a large staff was employed. The effect of the Bill in its present form was to cut out a large number of officers in the asylum service from its benefits as well as out of pensions. The Bill did not extend its advantages to all the servants employed in asylums, and it was the desire of the County Council that all the officers should share in its benefits. He pointed out that all officers were exposed to the risk of being injured by patients. Even members of the clerical staff were brought into contact with the patients when visiting the wards, and a special engineer told off by the London County Council to the asylums was often, in the course of his duties, exposed to attacks. Yet none of these officers would enjoy the benefits of the Bill. He submitted that as the Bill was modelled on the Poor Law Officers' Superannuation Act, which did not make exceptions, it should be extended to all officers employed. After a lengthy discussion these amendments were finally withdrawn.

IN the course of the debate on the other clauses, the Lord Chancellor said the Government supported the Bill, though it was purely a private Bill. Lord Belper moved a further amendment to limit the provisions of the Bill to servants or officials over fifty-five years of age who had completed twenty years' full service, which was finally accepted, after the Archbishop of Canterbury had pleaded vigorously for greater generosity. A second amendment making the sub-section relating to officers of the second class, was negatived, but the following new sub-section, moved by Lord Clifford, was agreed to:—(4) Where an established officer or servant of an asylum is injured—(*a*) in the actual discharge of his duty; and (*b*) without his own default; and (*c*) by some injury specifically attributable to the nature of his duty; and is permanently incapacitated from asylum duty as the result of such injury—the visiting committee of the asylum may grant to him such gratuity or special superannuation allowance as they may consider reasonable. The other clauses were adopted, with a few trifling alterations, and the Bill finally reported for third reading.

---

ONE of the most interesting papers read before the last General Congress of the American Association dealt with the hospital considered from the patient's point of view, and was the work of Dr. Thompson, the professor of medicine of the Cornell University. In opening the discussion, Dr. Thompson pointed out that while during recent years a great deal of attention had been paid to the administrative and scientific side of hospital work, comparatively little had been done in elucidating the point of view of the hospital patient. The average patient has the moral right to expect not only the best medical and surgical treatment, but what may be called environmental treatment and "consideration for himself as an individual, and not as a mere unit, lost in inflexible routine." Many hospital wards are mere noisy dormitories, where the patients, often with every variety of ailment, are herded together under identical conditions, with no adaptation whatever to their individual needs. Patients who need quiet and absolute rest—Dr. Thompson instanced enteric fever patients—are distressed by attendants, nurses, and doctors passing constantly in and out, and it is really remarkable, when one comes to think of it, what an amount of walking is done in the average hospital ward. Dr. Thompson, with the scientist's—especially the medical scientist's—love for the statistical, attached pedometers to three of his ward nurses, and found that each of them did four miles marching up and down the ward during her period of day duty alone!

---

MANY of Dr. Thompson's strictures call to mind some of the complaints which we have heard patients make in our own metropolitan hospitals, and some of his queries are therefore pertinent, and deserve attention. "Do we ever provide," he asks, "a place for the poor old woman, with chronic bronchitis and emphysema, to sit and warm her blue toes and be really comfortable as she would be in an easy chair at home? Sometimes there is a soulless radiator, but more often she has the exquisite privilege of sitting in a stuffy ward, kept at the same temperature day and night, and gazing at a hole in the wall far above her head, where some humanitarian architect has seen fit to place a hot-air flue, whereby she could not warm her feet if she stood on her head." Further on he draws attention to the absurd balconies and roofs of so many new and alleged up-to-date hospitals, and, a much more important point, to the idiosyncrasies of nursing. How often is the patient treated as a case instead of as a person by his nurses! He may have three separate nurses attending him in the course of one day—in one case, Dr. Thompson tells us he had seventeen different nurses in three weeks—and he is disturbed and fussed over whenever there is a change of nurses.

---

ALL these observations are doubtless very true, and Dr. Thompson does well to bring them before those interested in the welfare of hospitals. In America, unfortunately, the older Continental method of treating patients—a method which, we are glad to say, is dying out even in its home, the Public Clinic—is in vogue in some hospitals. How many patients "could a tale unfold" of their experiences under that system—a horribly brutal one—did they choose to do so, or, what is more to the point, did the hospital authorities choose to listen to them? In this country the treatment of patients is less of iron, and less ironic. All the same, there is room for improvement in our methods. We doubt very much, for example, if the Borough or Whitechapel old lady with chronic bronchitis and emphysema would have had that easy chair that Dr. Thompson conjures up in her garret at home, and probably she would find the cheerless heating of a Plenum-ventilated ward a welcome change from the more cheerless, though better ventilated, slum basement. It would be well if hospital administrators and superintendents spent a small portion of their time each week in going round their wards and talking to convalescent patients in order to find out the latter's opinions on their treatment. Perhaps they may have to listen to much foolish adulation, and more foolish objections, but in the sum of all expressions of opinion, many good hints may be hidden which are worth consideration.

---

THE modern hospital cannot hold itself complete as an institution for the scientific care of the sick unless it is fitted with a complete *x*-ray equipment. The question of the proper and efficient fitting up of the *x*-ray room is therefore a matter which demands the attention of every board of management. In large institutions, which possess a good income, that question is not of so much importance as it is in the case of small cottage hospitals, where the initial expense of fitting up a proper rœntgenological examining-room has to be carefully considered. For that reason it is desirable that the smaller institutions which have had such room in working during the past six or ten years should give detailed reports as to the cost and results of the work. Comparative tables of expense entailed by such work should prove very helpful to all connected with hospitals. At present we believe that there is a good deal of waste both in money and results in these *x*-ray departments. During the last few years, for example, a vast amount of very valuable clinical data has accumulated which would bear careful colation and precis. We shall be glad to hear what our readers have to say on these points, and to publish details of cost of upkeep and installation of *x*-ray apparatus in cottage hospitals if those readers who have had experience of the work will furnish us with them.

